# Negative index metamaterial at ultraviolet range for subwavelength photolithography

**DOI:** 10.1515/nanoph-2022-0013

**Published:** 2022-03-15

**Authors:** Qijian Jin, Gaofeng Liang, Weijie Kong, Ling Liu, Zhongquan Wen, Yi Zhou, Changtao Wang, Gang Chen, Xiangang Luo

**Affiliations:** Key Laboratory of Optoelectronic Technology & Systems (Chongqing University), Ministry of Education, and College of Optoelectronic Engineering, Chongqing University, Chongqing 400044, China; State Key Lab of Optical Technologies on Nano-fabrication and Micro-engineering, Institute of Optics and Electronics, Chinese Academy of Sciences, Chengdu 610209, China

**Keywords:** negative index metamaterial, plasmonic waveguide, subwavelength photolithography

## Abstract

A negative index metamaterial (NIM) at ultraviolet range is constructed with stacked plasmonic waveguides. Based on the waveguides performing antisymmetric modes, the negative refractions of both wavevector and energy flow are realized when a TM-polarized light with a wavelength of 365 nm incidents on the plane of the layers. It is proved that the NIM could be introduced into subwavelength photolithography for extending working distance. Both theoretical and experimental results indicate that the patterns with a feature size of 160 nm can be reproduced in photoresist with a 100 nm-thick air working distance. Moreover, arbitrary two-dimensional patterns with a depth reach 160 nm can be obtained without diffraction fringe by employing a nonpolarized light. This design gives new insights into the manipulation of light. The improved working distance, well-shaped patterns over large area present an innovative method for improving subwavelength photolithography.

## 1 Introduction

Nowadays, photolithography is one of the most important ways in fabricating integrated circuit and nano devices [1, 2]. In particular, projection photolithography method can produce arbitrary patterns over large area with a single exposure process [3]. However, the resolution of traditional photolithography is always blocked by the so-called diffraction limit due to the wave nature of light [4]. Even though the resolution can be improved remarkably by decreasing the working wavelength, using immersion lithography, or multipatterning process, these technologies would involve much more complexity and cost. Hence, new cost-effective methods are needed to take the technique past the diffraction limit without huge expense. Recently, various techniques have been developed for pushing photolithography to higher resolution performance, such as stimulated emission depletion lithography, absorbance-modulation optical lithography, photothermal lithography [5], [6], [7]. By contrast, near-field photolithography is a straightforward way to produce subdiffraction limited patterns by collecting the evanescent waves before they decay [8]. In this instance, plasmonic lithography has been developed attractively [9, 10]. This method would enhance evanescent waves by exciting surface plasmons to compensate the decay and then lead to a super-resolution pattern. However, the photoresist (PR) has to be close contacted to the plasmonic devices, while, direct-write lithography systems [11], [12], [13] do not meet the requirement of obtaining arbitrary patterns with a single exposure. Although some special approaches, such as hyperbolic metamaterials and waveguide cavities are investigated to adjust the evanescent waves in more degrees of freedom [14], [15], [16], [17], [18], the overly short working distance (WD) and quite shallow pattern depth still obstruct the practical application of this exciting lithography scheme.

Interestingly, the negative index metamaterials (NIMs) has been studied many years due to its inherent perfect lens properties [19]. The light obeys Snell’s laws of refraction at the surface as light inside the NIMs, and forms a negative angle with the surface normal. Then an image of light source would be shaped by refocusing light on the other side of the NIMs, which is predicted to obtain subwavelength focusing with resolution well below the diffraction limit. More importantly, the WD could be extended theoretically by increasing the thickness of the NIM [20]. Although there is no material with both negative permittivity and permeability in nature, the negative refractive index could be realized in microwave or even infrared regions by constructing artificial metamaterials [20, 21]. However, it is difficult to achieve an acceptable negative refractive index at ultraviolet frequency because of substantial resonance losses and fabrication difficulties [22, 23]. Interestingly, plasmonic waveguide-based metamaterials can achieve a negative refractive index at high frequencies [24, 25]. It is reported that the negative refraction at the dimension of perpendicular to the plane of the layers could be achieved by employing strongly coupled plasmonic waveguides with symmetric modes [26, 27]. However, the excessive loss from the thick metal makes it not suitable for subwavelength photolithography.

Herein, we propose a subwavelength photolithography design by introducing a special NIM, which is constructed with stacked plasmonic waveguides but perform antisymmetric modes. Besides being achieved in-plane, the negative refraction of both wavevector and energy flow would be also achieved when an ultraviolet transverse magnetic (TM)-polarized light incidents on the plane of the layers. The experimental results, along with numerical calculations, show that the air WD could be greatly improved in the exposure process, and the subwavelength patterns could be produced in PR with well-shaped morphologies. Furthermore, arbitrary two-dimensional patterns over large area could be obtained by a single exposure with a nonpolarized light. This NIM-based design presents a more practical method for improving subwavelength photolithography.

## 2 Principle and design

**Figure 1:**
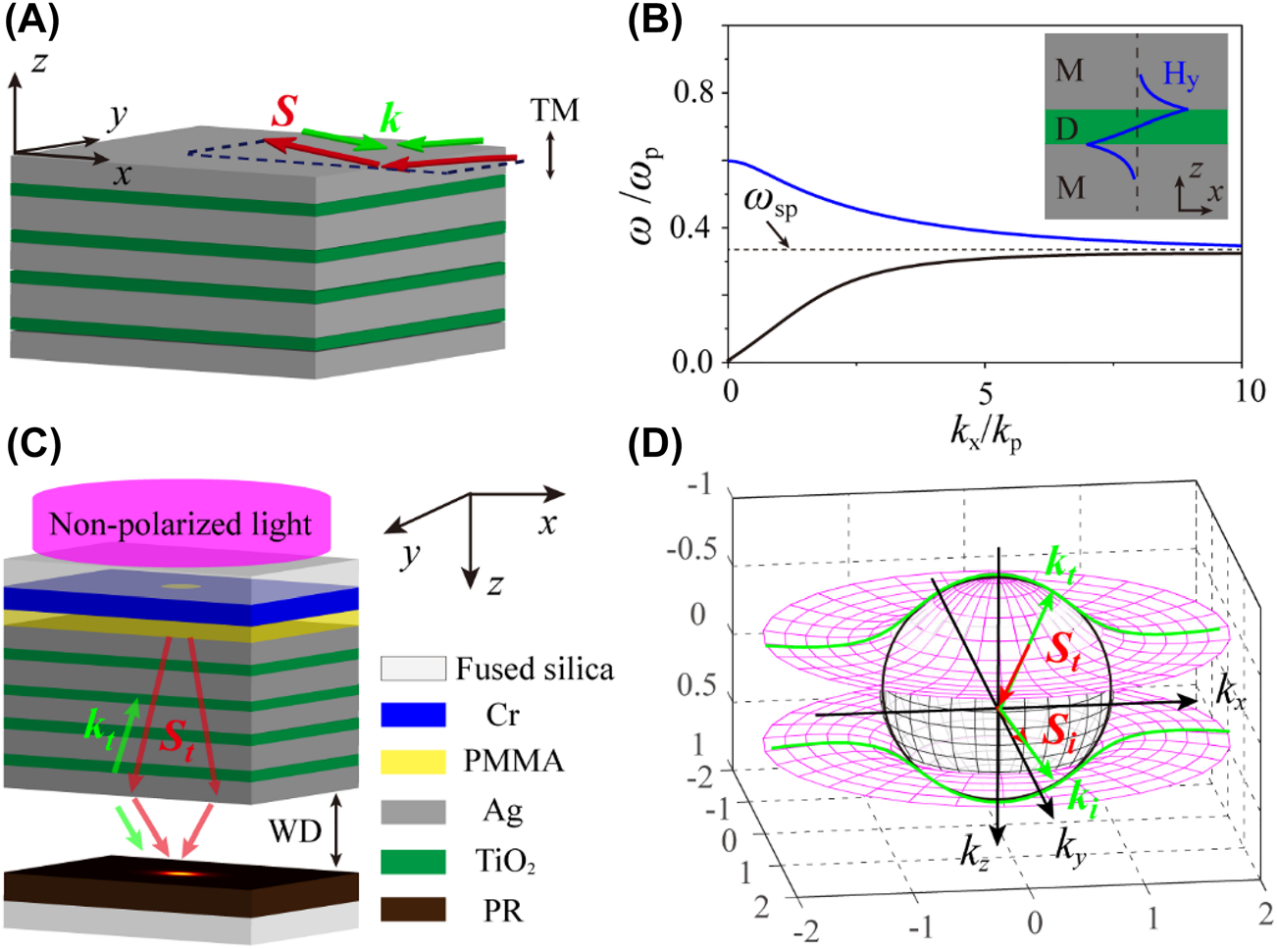
Design of a NIM for photolithography. (A) Schematic of plasmonic waveguide-based NIM multilayer. The dashed lines indicate the incident plane of a TM-polarized light parallels to the layers. Red and green–blue arrows depict the directions of ***S*** and ***k***, respectively. (B) Dispersion relations for modes in an MDM waveguide with *h*_d_ = 19 nm and *ε*_d_ = 8.52, where *k*_p_ = *ω*_p_/*c*. The corresponding structure and magnetic field distribution Hy along the *z* axis are shown in the inset. (C) Schematic of the NIM-based lithography design. (D) Three-dimensional plots of EFCs for the plane wave with a wavelength of 365 nm in NIM slab and in free space. ***S***_***i***_ and ***S***_***t***_ show the incident and transmitted time-averaged Poynting vectors, respectively; ***k***_***i***_ and ***k***_***t***_ give the incident and transmitted wavevectors, respectively.

Generally, NIMs are a kind of medium possess simultaneously negative permittivity, *ε* < 0, and negative permeability, *μ* < 0, which then leads to a negative refractive index, *n* < 0. The characteristics of plane waves in this medium can be described by the wavevector ***k*** and the time average Poynting vector ***S***, which, respectively, give the directions of phase velocity ***V*_p_** and energy flow, while, the direction of ***S*** could be further represented by the direction of group velocity ***V*_g_** in the absence of loss. In this case, the medium, originally named ‘left-handed’ material, supports propagating solutions whose ***V*_p_** and ***V*_g_** are antiparallel. It means the signs of ***k*** and ***S*** are opposite (***k*** • ***S*** < 0) [28]. Unlike traditional materials, the NIMs are often composited of noble metals (M) and dielectrics (D) planar films in the visible and ultraviolet ranges [29], [30], [31]. As shown in Figure 1A, the NIM structure is a stacked multilayer, and each unit cell has an MD layer sequence. The arrows depict the directions of ***k*** and ***S***, performing negative refraction and backwards phase propagation. However, such NIM is generally regarded as a purely two-dimensional isotropic, in other words, the negative refractive only could be observed in-plane [24], [25], [26], [27].

To be used in photolithography, the negative refraction need be extended to another dimension normal to the plane of the NIM multilayers. Here, the proposed NIM structure is consist of alternating Ag and TiO_2_ layers, which are employed to form plasmonic waveguides stacked in the *z* direction, and each unit cell has an MDMD layer sequence (40, 19, 45, and 19 nm, respectively). The individual film thicknesses are optimized to achieve left-handed response and admissible transmittance in the ultraviolet band (Supplementary Materials S1). It is worth noting that the transmittance could be enhanced by employing a material with low absorbtion, e.g. HfO_2_ (*n* ≈ 2.2) [32]. However, the NIM multilayer should be designed comprehensively. A dielectric with high refractive index and a metal with low loss would present an NIM with attractive performance. Figure 1B gives the dispersion curves of the propagating modes in the designed plasmonic waveguides, which is computed with transfer matrix method by using $\epsilon_{m}=1-\omega_{p}^{2}/\omega^{2}$ and *ε*_d_ = 8.52. The left-handed response acquires a negative ***V***_g_ (i.e., d*ω*/d*k* < 0), which is accomplished by the plasmonic waveguide in a frequency range between the volume plasma frequency of the metal *ω*_p_ and the surface plasmon frequency of the metal-dielectric interface (i.e., *ω*_sp_ < *ω* < *ω*_p_). Hence, the ***k*** and ***S*** are counterpropagating without considering the absorption, meaning the NIM can perform negative refraction in-plane. In addition, the waveguide supports antisymmetric mode because of the dielectric thickness $h_{d}<\left(\pi c/\omega_{p}\right)\sqrt{\left(1+\epsilon_{d}\right)/\epsilon_{d}}$ [24], which is present by the magnetic field *H*_*y*_ in the inset. More importantly, this NIM also exhibit negative refraction when a TM-polarized light illuminates on the plane of the multilayers. It could be observed that the phase front in TM condition is refracted in a negative angle and propagated with a backward direction (Supplementary movies 1 and 2). Meanwhile, the direction of ***S*** inside the multilayer is antiparallel to the ***V***_p_, which is a signature of NIM. These fresh evidences state clearly that the proposed NIM with antisymmetric mode in waveguides could be used in subwavelength lithography.

The diagram of the designed photolithography system is shown in Figure 1C. A plane wave radiated from a mercury lamp (*i*-line) normally incidents on the Cr mask from the top side. The slots of the mask are flatted by coating poly(methyl methacrylate) (PMMA), which also works as an index-matching layer [10, 15, 33]. The NIM multilayer includes two MDMD layered unit cells and an additional Ag coupling layer. The waves transmitted through the multilayer and 100 nm-thick air WD are regathered into the PR, which is supported by another substrate.

To illustrate the features of the NIM more clearly, the three-dimensional equi-frequency contours (EFCs) are calculated by using *ε*_m_ = −2.26 + 0.46i and *ε*_d_ = 8.52 + 0.31i (Figure 1D). The EFCs map the angular dependence of the wavevector in the NIM and indicate the direction of the ***S*** (which is normal to the EFCs and points in the direction of its displacement as a function of frequency). It can be seen that the EFCs of the NIM resemble a sphere with a radius of approximately *k*_0_. The wavevector magnitude decreases for increasing frequency, indicating that the ***V***_g_ is oriented inwards. The red arrow confirms that the ***S*** is directed inwards (i.e., ***k*** • ***S*** < 0). For a plane wave incident from mask onto the surface of NIM, the transmitted backwards wave would have a ***k***_***t***_ (pointing towards the interface) and a ***S***_***t***_ (pointing away from the interface) on the same side of the normal as the incident light, which is inferred from the continuity of phase and conservation of energy [27]. The vivid description of phase fronts in this lithography system (normal incidence case) can be seen in Supplementary movie 3. It should be noted that ***k*_*t*_** and ***S*_*t*_** are not exactly collinear, which is mainly because the EFCs of the NIM is not a perfect sphere.

**Figure 2:**
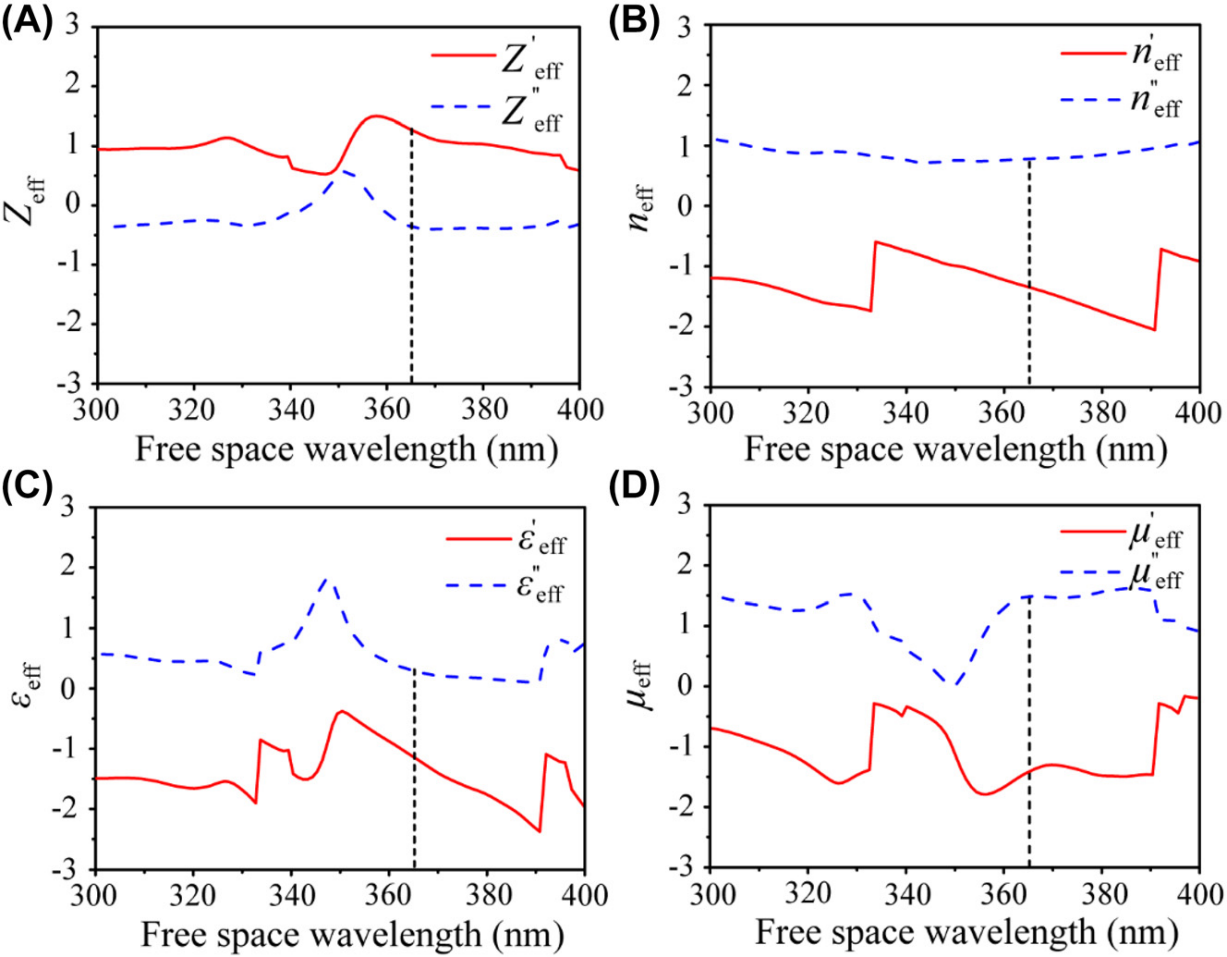
Effective parameters for the designed NIM in the wavelength range of 300–400 nm. (A–D) Corresponding real (′) and imaginary (″) parts of the retrieved effective relative impedance *Z*_eff_, index *n*_eff_, permittivity *ε*_eff_, and permeability *μ*_eff_.

Basically, the NIM multilayer could be considered as an equivalent negative refractive slab. To give a visualized parametric analysis and confirm the validity of negative index for the proposed NIM, we perform a parameter retrieval procedure using the commercial software FDTD solutions 2019. The NIM multilayer in air environment is normally illuminated by a broadband plane wave source with the wavelength ranging from 300 nm to 400 nm. After the steady state field distributions is obtained, the complex reflection *r* and transmission *t* coefficients are calculated by taking the ratios *r* = *E*_r_/*E*_0_ and *t* = *E*_t_/*E*_0_, where *E*_0_ is the complex electric field amplitude of the incident light, *E*_r_ and *E*_t_ are the reflected and transmitted complex electric field amplitudes, respectively. Then, *r* and *t* could be related to the effective relative impedance *Z*_eff_ and index *n*_eff_ by using standard inverted reflection and transmission parameter equations [34]. The effective relative permittivity *ε*_eff_ and permeability *μ*_eff_ are calculated through the relations *ε* = *n*/*Z* and *μ* = *nZ*. Figure 2 shows the resulting curves corresponding to the effective relative *Z*_eff_, *n*_eff_, *ε*_eff_, and *μ*_eff_ of the NIM multilayer. It can be seen that the real part of the extracted *n*_eff_ is negative over the entire spectral region. Specially, the real part of the *n*_eff_ is about −1.35 at the wavelength of 365 nm. Meanwhile, it shows a double-negative index composed of simultaneously negative real parts of the permittivity and permeability. Therefore, the proposed NIM not only performs negative refraction [35, 36], but also exhibits a true negative refractive index characterized by negative refraction and backwards phase propagation. These parameters are extremely consistent with the definition of NIM [37]. If the NIM multilayer is consider as an isotropic medium with *n*_eff_ = −1.35, the direction of light could be observed intuitively with ray-tracing method (Supplementary material S2). On the other hand, when a plane wave in free space with an incident angle *θ*_*i*_ passing through the boundary between air and NIM, the *n*_eff_ also could be estimated qualitatively by using Snell’s law, i.e., *n*_eff_ = sin*θ*_i_/sin*θ*_r_, where *θ*_r_ is the refraction angle of ***S*** inside the NIM. In our case, the calculated *n*_eff_ of the NIM is about −1.31 (Supplementary material S3), in agreement with the value derived from the parameter retrieval procedure.

**Figure 3:**
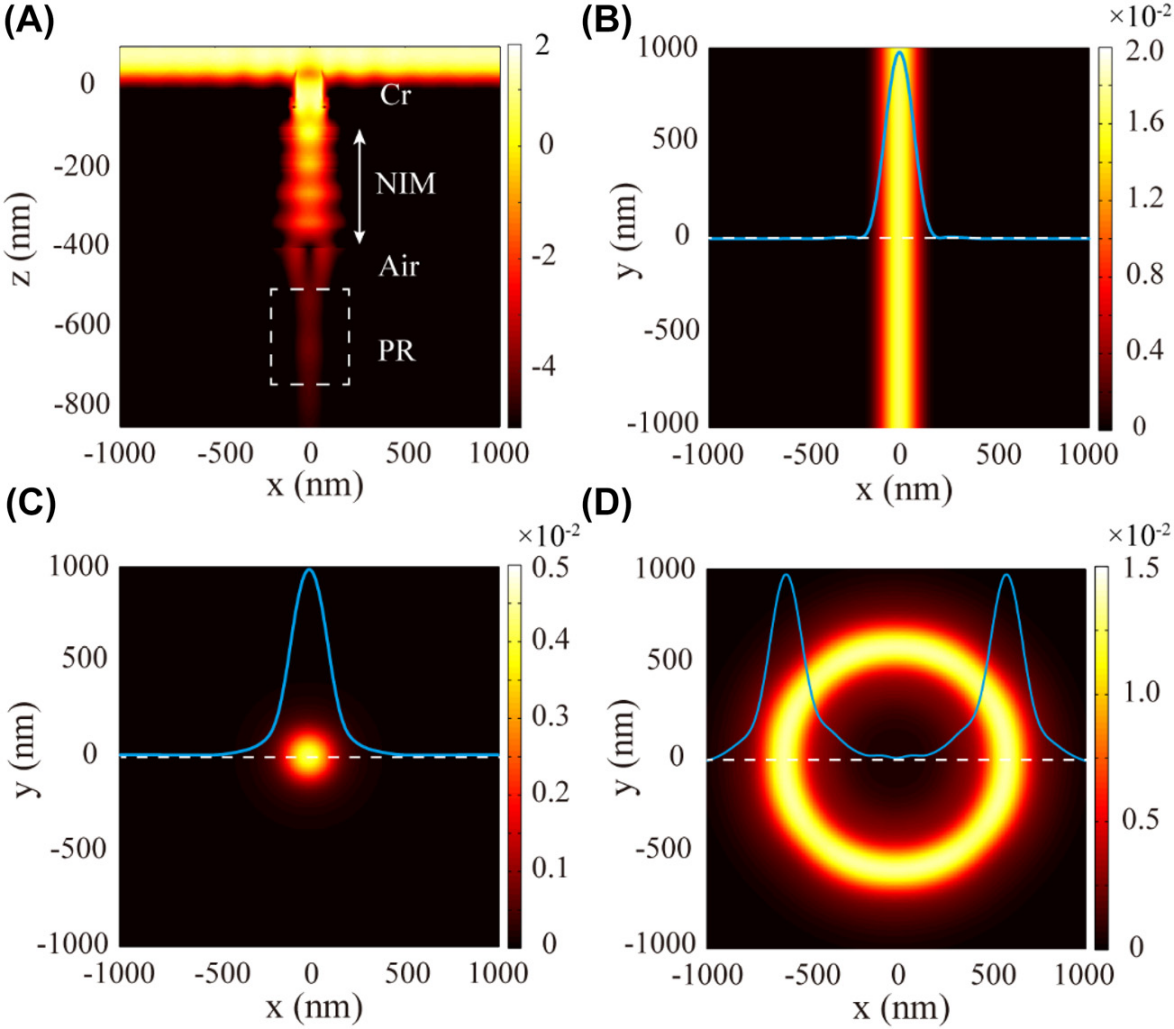
Simulated results of the NIM-based lithography. (A) Cross section of normalized intensity distribution in logarithm scale inside the NIM-based lithography design. (B–D) Normalized intensity distributions in *x*–*y* plane extracted from the middle position of PR. The corresponding mask patterns are straight slit, isolated aperture, and annular slit, respectively. The insets show the related intensity distributions along the dashed lines.

To verify the foregoing analysis, several typical mask patterns are employed to demonstrate the capability of the proposed photolithography system. Firstly, a straight slit with a width of 160 nm is prepared on the mask layer (*ε*_Cr_ = −8.62 + 9.23i) [38]. The normalized intensity distribution in *x–z* plane is given in Figure 3A. Obviously, when the mask is irradiated by a TM-polarized light with a wavelength of 365 nm, the diffraction waves could be excited and propagated efficiently in the NIM with negative refraction angles, which then are refocused after passing through the air WD. Because the realistic dispersion, loss would result in the NIM never meet the idealized conditions [39], the diffracted waves are refocused at different positions. Finally, a pattern is formed in the following PR layer (*ε*_PR_ = 2.59) [33] with a depth over 200 nm. The cross section taken from the middle position of PR layer shows the full width at half maximum (FWHM) of the straight line is ∼170 nm (Figure 3B).

Then, the masks changed into an isolated aperture with a diameter of 180 nm. Notably, to obtain standard two-dimensional patterns and imitate a nonpolarized light used in following experiments, the mask is illuminated by superposing two incoherent linearly polarized lights with vertical polarization. The normalized intensity distribution in PR shows a round dot with an FWHM of ∼220 nm (Figure 3C). The size expansion is mainly due to the adverse effect of TE-polarized components in hybrid lighting (Supplementary materials S4), which also can be seen in the annular slit mask case shown in Figure 3D. It presents that the annular slit with an outer diameter of 700 nm and an inner diameter of 500 nm is regularly imaged into the PR with an FWHM of ∼220 nm. Remarkably, the optical fields in PR have no any sidelobe or diffraction fringe, meaning the patterns could be produced with high fidelity, which is highly desirable in lithography. Admittedly, two-dimensional patterns also could be generated by illuminating with circularly polarized light [14, 40]. However, the nonpolarized lights radiated from a mercury lamp has apparent advantages in respect to convenience and robustness.

## 3 Fabrication and lithography results

**Figure 4:**
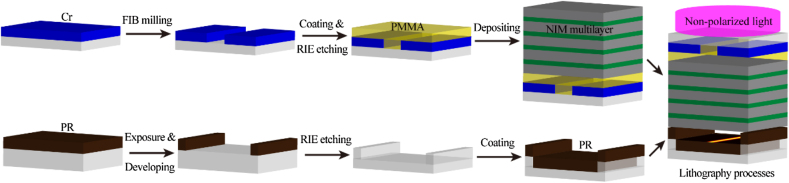
Schematic diagrams for the processes of sample fabrication and lithography.

The key fabrication and exposure processes of the NIM-based lithography is shown in Figure 4. Crucially, the mask is flatted by the PMMA, which give a planar surface for depositing the NIM multilayers. The 100 nm-thick air WD is realized by fabricating a sunken step on the PR substrate around the patterning areas. More importantly, the nonpolarized light radiated from a high pressure mercury lamp is used directly for exposure. All the patterns are produced with a single exposure process. The relevant details are described in the Experimental Section.

**Figure 5:**
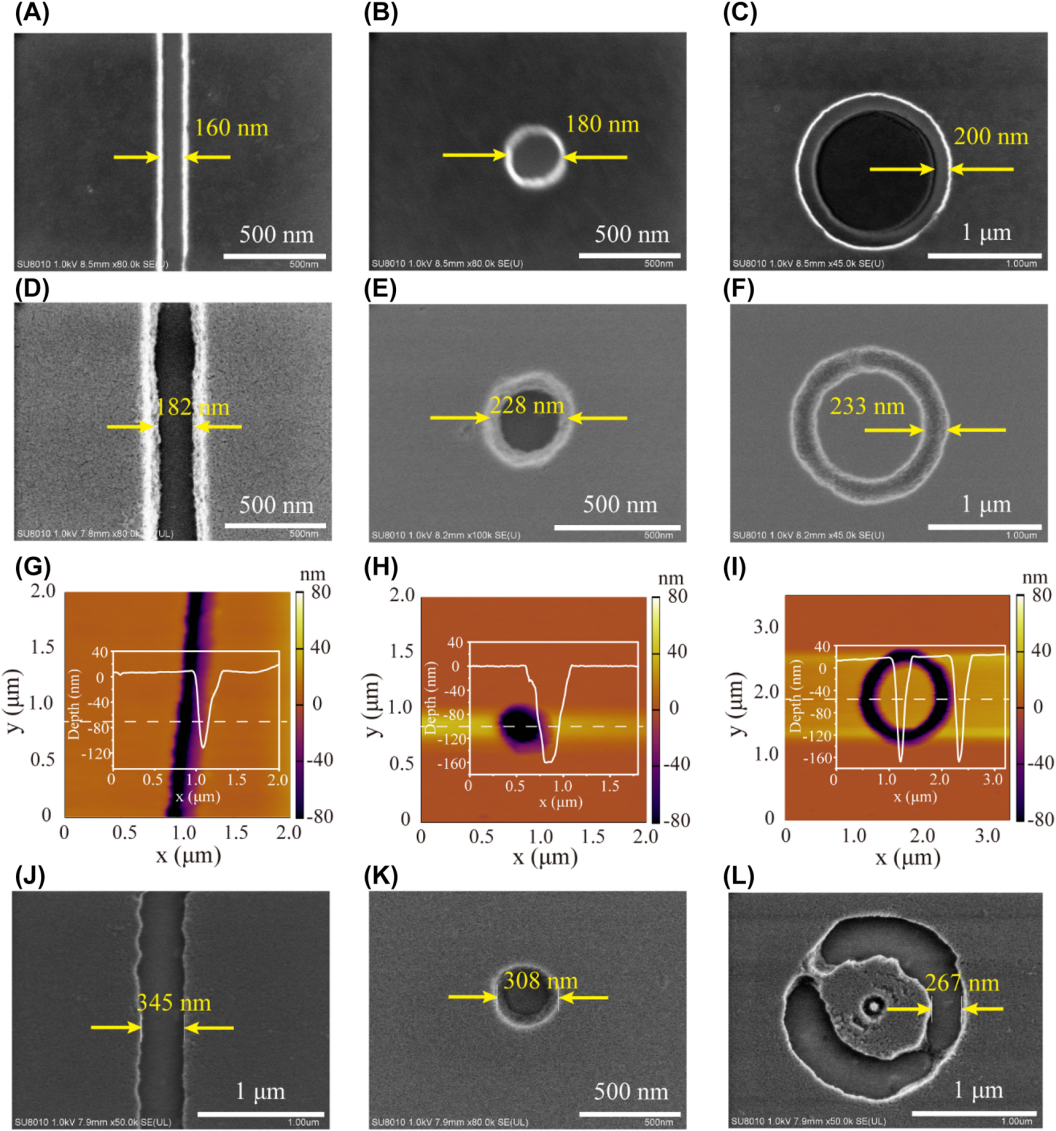
Experimental results of typical mask patterns in lithography with/without NIM multilayer. (A–C) SEM images of the masks with a straight slit, isolated aperture, and annular slit, respectively. (D–F) Corresponding SEM images of the patterns formed in PR. (G–I) Corresponding AFM images of the PR patterns. The insets present the depth profiles along the dashed lines. (J–L) Corresponding SEM images of the PR patterns in control experiments.

Figure 5 shows the experimental results of the aforementioned three different masks. The pictures in left column are related to the straight slit condition. Figure 5A is a scanning electron microscopy (SEM) image of the mask. As expected, after transmitted through the NIM multilayer and air WD, the waves generate an identical pattern in PR but the width is ∼180 nm (Figure 5D), which is close to the simulated result in Figure 3A. The difference could be ascribed to the broadband emission from the mercury lamp (Supplementary Materials S5). The morphology of the pattern is measured by atomic force microscopy (AFM) and shown in Figure 5G. Clearly, the depth of the pattern exceeds 120 nm, which surpasses almost all reported results based on plasmonic lithography [14, 40, 41]. More importantly, the neat patterning area shows no diffraction fringe around the slit. Same performances can also be seen in another two mask cases (corresponding pictures in Figure 5B, E, H and Figure 5C, F, I), where both the isolated aperture and annular slit are successfully imaged into PR with well-shaped morphologies. The widths of the two patterns in PR are ∼230 nm, and the depths reach 160 nm. Actually, if the adverse effect of TE-polarized components is eliminated by illuminating with a radially polarized beam, and using a light source with narrow wavelength band, the FWHM of a circular or annular pattern could be dramatically shrunk [42].

The control experiments are also carried out. Without the NIM multilayer, the light intensity in PR could be enhanced significantly, resulting in less exposure time. However, the light beams would diverge greatly as the propagating distance increases. The patterns in PR are expanded accordingly (Supplementary Materials S6). As observed in Figure 5J–L, the widths of the straight slit, isolated aperture, and annular slit are 345, 308, and 267 nm, respectively, which are much larger than the NIM-based results. The rough edges and tiny ring in the central of the annular slit proves that unwanted interferences are happened during the imaging process.

**Figure 6:**
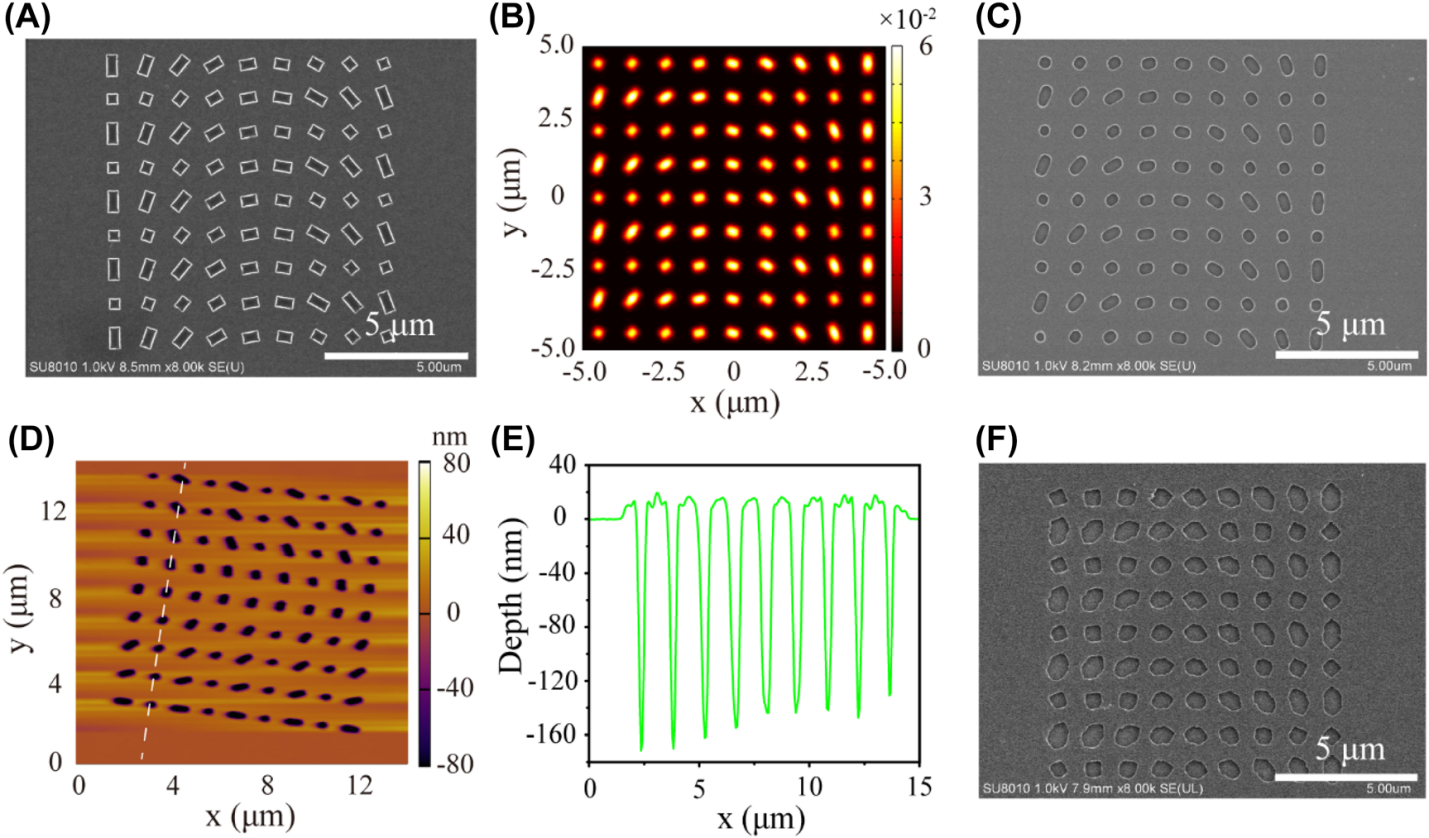
Experimental results of a mask with patterns over large area. (A) SEM image of a mask constructed with aperiodic rectangles. (B) Normalized intensity distributions extracted from the position of 100 nm-depth PR. (C, D) SEM and AFM images of the pattern formed in PR. (E) Corresponding depth profile along the dashed line in (D). (F) SEM images of the PR patterns in control experiment.

The proposed subwavelength lithography system can also fabricate arbitrary patterns over large area with a single exposure process. As illustrative examples, typical optical metasurface structures [43], [44], [45] are used to demonstrate an economical and effective method in fabrication. Figure 6A shows an SEM image of rectangles array mask. The widths of all rectangles are 400 nm, while the lengths vary from 400 nm to 800 nm. Benefited from negative refraction of the NIM, the waves diffracted from the mask could be regathered and produce well-shaped patterns in PR (Figure 6B–E). The patterns with neat morphologies indicates the disordered diffraction and intersect interference maybe not happened or affect the imaging process (more results in subwavelength sizes can be seen in Supplementary materials S7). Whereas the morphologies of the patterns in the control experiments present obvious distortions (Figure 6F). Same performances can be seen in the conditions of annular slits arrays and *V*-shaped antennas (Supplementary material S8 and S9). Regrettably, the phenomena of corner rounding can be seen in the results of NIM-based lithography, which reveals it still suffers from the optical proximity effect. However, this problem could be resolved distinctly by combining the technology of optical proximity correct [46].

## 4 Conclusions

In conclusion, an NIM is designed based on alternating Ag/TiO_2_ multilayer with antisymmetric waveguide mode. It is proved that the negative refraction of both wavevector and energy flow can be realized when a TM-polarized light incident on the plane of the layers. Furthermore, the NIM could be introduced into subwavelength photolithography to extend the WD and produce well-shaped arbitrary patterns over large area. In the case of 100 nm-thick air WD, subwavelength patterns with a depth reach 160 nm could be obtained by illuminating with a mercury lamp. This strategy gives new insights into nanofabrication. The principle is also meaningful for studying optical imaging, reversed Doppler effect, compact on-chip devices, etc.

## 5 Experimental section

First of all, a fused silica substrate is boiled in H_2_SO_4_:H_2_O_2_ (3:1) solution and ultrasonic cleaned in acetone and deionized water successively. The Cr film with a thickness of 60 nm is deposited on the substrate by Magnetron Sputtering (DE500, TE technology) with a power of 400 W in radio frequency mode. The deposition rate is 0.5 nm/s at a base pressure of 6.0 × 10^−5^ Pa. The mask patterns used in this work are milled on the Cr film by Focused Ion Beam (Helios Nanolab 650, FEI Company, @30 kV accelerating voltage). A PMMA layer with a thickness of 80 nm is spin-coated on top of Cr mask and baked for two and a half hours at 120 °C. Then, the PMMA layer is etched by reactive ion etching with O_2_ to get a planar surface, and the remained thickness is about 28 nm. The 9 layers of Ag/TiO_2_ multilayer are alternatively deposited on the PMMA surface by magnetron sputtering without breaking vacuum. The optimized sputtering conditions include an Ar gas flow rate of 8 sccm, a base pressure of 5.0 × 10^−5^ Pa, a power of 200 W in radio frequency mode for TiO_2_ and 100 W in direct current mode for Ag. The deposition rates for Ag and TiO_2_ are 0.56 nm/s and 0.042 nm/s, respectively. The permittivities of Ag and TiO_2_ used in main text are measured by Ellipsometer (SE 850, SENTECH).

In addition, the air WD is realized by fabricating a sunken step on the PR substrate around the patterning area. Firstly, the AR-3100 positive PR (ALLRESIST GmbH, Strausberg) with a thickness of ∼1000 nm is spin-coated onto a substrate and baked for 5 min at 100 °C. A sunken step with a diameter over 10 mm is fabricated by traditional photolithography. Then the step is transferred to the substrate by reactive ion etching with CH_3_F_3_. And the etching depth is ∼100 nm. The acetone is used to remove the residual PR. At last, The AR-P 3170 positive PR is spin-coated onto the substrate and baked for 5 min at 100 °C. The PR thickness at the sunken step area is ∼160 nm measured by a surface profiler.

In the exposure process, the PR/substrate is in conformal contact with NIM/mask/substrate with the aid of air pressure (∼0.8 MPa). All the masks are normally illuminated by a nonpolarized light with a central wavelength of 365 nm (*i*-line) radiated from a mercury lamp. The exposure intensity and time are set to be 0.22 mW/cm^2^ and 300–400 s, respectively. Then, the PR layer is developed for 20 s in a mixed solution of AR 300–35 and deionized water with a volume ratio of 1:1, rinsed with deionized water and dried by N_2_ gas in order.

## Supplementary Material

Supplementary Material

Supplementary Material
