# Supplementary material for: Negative index metamaterial at ultraviolet range for subwavelength photolithography
Source: Nanophotonics. 2022 Mar 15;11(8):1643–51. doi: 10.1515/nanoph-2022-0013 (PMC11501468; doi:10.1515/nanoph-2022-0013)
Supplement: Supplementary file 1 — Supplementary Material [file j_nanoph-2022-0013_suppl_001.docx]

**Supplementary material**

Negative index metamaterial at ultraviolet range for subwavelength photolithography

*QiJian Jin^1,2^, GaoFeng Liang^1,*^, Weijie Kong^2^, Ling Liu^2^, ZhongQuan Wen^1^, Yi Zhou^1^, Changtao Wang^2^, Gang Chen^1^, and Xiangang Luo^2,^*^*^

^1^ Key Laboratory of Optoelectronic Technology & Systems (Chongqing University), Ministry of Education, and College of Optoelectronic Engineering, Chongqing University, Chongqing 400044, China

^2^ State Key Lab of Optical Technologies on Nano-fabrication and Micro-engineering, Institute of Optics and Electronics, Chinese Academy of Sciences, Chengdu, 610209, China

*Corresponding authors. E-mail: lgf@cqu.edu.cn; lxg@ioe.ac.cn

**S1. Transmittance of the NIM multilayer in ultraviolet range**

The most recently work about NIM at ultraviolet range (*λ*=363.8 nm) was reported in 2013 [1]. The structure, based on stacked plasmonic waveguides, yields an omnidirectional left-handed response for transverse magnetic polarization characterized by a negative refractive index. It performs symmetric waveguide mode, which is designed based on repeated MDMDM layered unit cells. However, it is unsuited in lithography because the excessively loss from thick metal would dramatically increase the exposure time. When it is illuminated vertically by an ultraviolet plane wave of free-space wavelength 363.8 nm, the transmittance is only 0.0167 (Figure S1A), which is calculated by using the commercial software FDTD solutions 2019. Although the negative power refraction of the ultraviolet light could be observed over a broad range of incident angles spanning 10°~60°, the transmittance at all angles does not exceed 0.023.


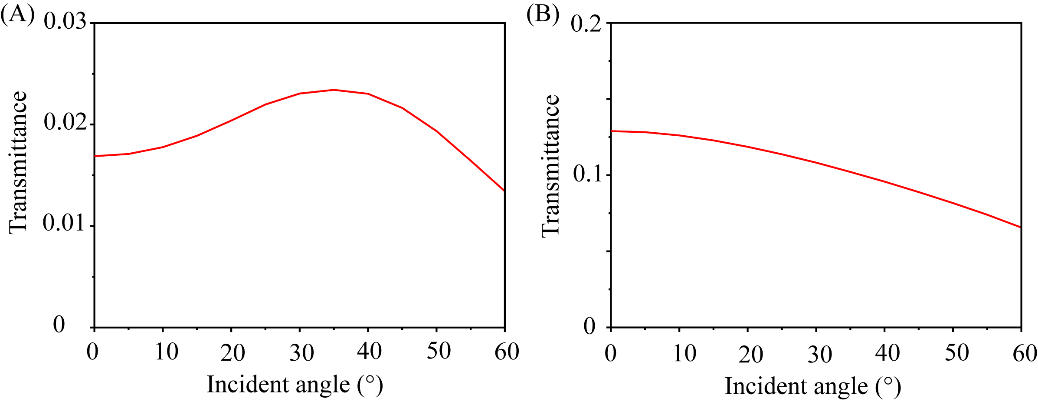


Figure S1. (A) The transmittance of the NIM reported in reference [1] with different incident angle. (B) The transmittance of the NIM proposed in main text with different incident angle.

In contrast, the NIM proposed in main text performs antisymmetric waveguide mode, which is designed based on repeated MDMD layered unit cells. When a light with a wavelength of 365 nm incidents vertically on the NIM multilayer, the transmittance is 0.129 (Figure S1B), which is ~8 times of the reported NIM. Even the transmittance decreases as the incident angle increases, the transmittance is 0.06 at 60°. In addition, the transmittance of this NIM exceeds that of most metal-dielectric multilayers used in photolithography [2, 3]. Therefore, we declare it achieved an admissible transmittance for producing subwavelength pattern in lithography.

Actually, it is very important to choose right constituent layers for designing a NIM structure, especially in the ultraviolet range (*λ*=365 nm). In the main text, Ag (*ε_Ag_* = -2.26+0.46*i*) is chosen as the metal component because of its high transmittance. While, TiO_2_ (*ε_TiO2_* = 8.52+0.31*i*) is employed as the dielectric component due to its high refractive index. It is indicated that a large dielectric index ensures *ω_sp_* is relatively small, extending the operating frequency window to the ultraviolet spectrum [4]. In addition, the multilayer composed of Ag and TiO_2_ has an isotropic phase index, which results in a circular equi-frequency contour.

It should be note that HfO_2_ film exhibits a high refractive index (𝑛 ≈ 2.2) and a negligible extinction coefficient (*k* ≈ 0), and possesses a higher optical transmittance (> 90 %) than TiO_2_ material in the ultraviolet range [5, 6]. Therefore, HfO_2_ material would be a competitive material to construct novelty metamaterials and metasurfaces, and could potentially be used to construct NIM structure as the dielectric component. However, its refractive index is much lower than that of TiO_2_ material at the wavelength of 365 nm. The NIM multilayer should be designed comprehensively. A dielectric with high refractive index and a metal with low loss would present a NIM with attractive performance. Thus, HfO_2_ film may perform not well as TiO_2_ film in the NIM design.

**S2. Simulation of light passing through a negative index slab with ray-tracing method**


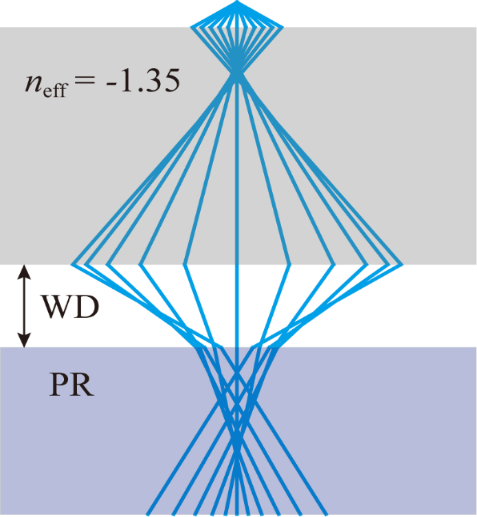


Figure S2. Simulation of light passing through an isotropic slab with ray-tracing method. WD denotes the working distance in air environment.

In main text, the parameter retrieval procedure indicates the effective refraction index *n*_eff_ of the designed negative refraction metamaterial (NIM) is -1.35. If the NIM multilayer is considered as an isotropic slab, the direction of light could be observed intuitively with ray-tracing method. As shown in Figure S2, a point optical source is placed in front of the slab, the rays are diverged gradually as the distance increases. Once transmitted to the surface of the slab, the rays would refracted with a negative angle, but they still obey the Snell’s laws of refraction. In addition, double focusing effect is revealed by this simple ray diagram. After passing through the slab, all rays would converge into the photoresist (PR) with an air working distance (WD). Therefore, the designed NIM could be used to produce subwavelength patterns without diffraction fringe.

**S3. Simulation of an off-axis incident light transmitted in the NIM**

Figure S3 gives a cross section of an off-axis incident light transmitted in the proposed NIM, which is simulated in commercial software FDTD Solutions 2019. When the light impinges on the mask with an incident angle of 30°, both phase velocity ***V_p_*** and energy flow, represented the wave vector ***k*** and time-averaged Poynting vector ***S*** respectively, have a negative refraction angle after the light transmitted into the NIM multilayer. That also happened when the light gets out the NIM multilayer.


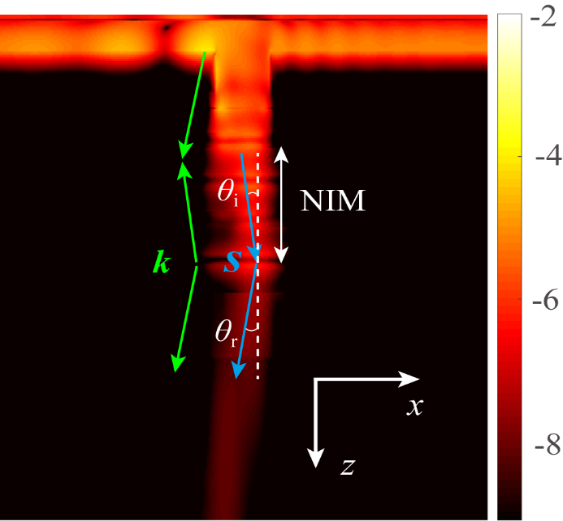


Figure S3. Normalized intensity distribution in logarithm scale extracted from the NIM-based lithography system. The light incidents on the mask with an angle of 30°. *θ_i_* and *θ_r_* are corresponding incident and refraction angles for plane wave transmitting from the NIM to air space.

The effective refractive index *n_eff_* could be estimated qualitatively by using Snell's law and the observed refraction angle of ***S*** inside the NIM structure. As depicted in Figure S2, if a plane wave with an incident angle *θ_i_* is transmitted from the NIM to air space, and the refraction angle *θ_r_* could be demarcated, the *n_eff_* could be calculated directly with sin*θ_r_* / sin*θ_i_*. Here, we use the E-ruler software to measure the angles. It shows that the incident angle is about 9.5°, and the refraction angle is about -12.5°. Thus, the calculated *n_eff_* of the NIM is -1.31, which is approach to the value derived from the parameter retrieval procedure in main text.

**S4. The effect of the TE-polarized components in subwavelength lithography**

In this work, the NIM is designed based on plasmonic waveguides, which is constructed with metal and dielectric planar films. It is widely known that evanescent waves with high spatial frequency could be coupled with surface plasmon polaritons, which is a cluster oscillation of electronics and can only be excited by TM-polarized light. With the evanescent wave involved, the subwavelength mask could be imaged into PR with high resolution. However, the TE-polarized light cannot perform this function, the diffracted waves would diverge gradually as the distance is increased, leading to expanded patterns accordingly. These analyses could be supported by Figure S4, where the mask is a circular aperture with a diameter of 180nm. When a linearly polarized light illumines the mask with the electric field component along the *x*-axis (i.e. TM polarized in *x*-axis, but TE polarized in *y*-axis), an elliptical pattern is formed in PR with a FWHM(x) of ~200 nm, but with a FWHM(y) of 290 nm. If the light source is superposed by two incoherent linearly polarized lights with vertical polarization, as stated in main text, the pattern is circular and its FWHM is ~220 nm, which is larger than FWHM(x), but smaller than FWHM(y). Therefore, the size expansion could be ascribed to the adverse effect of TE-polarized components.


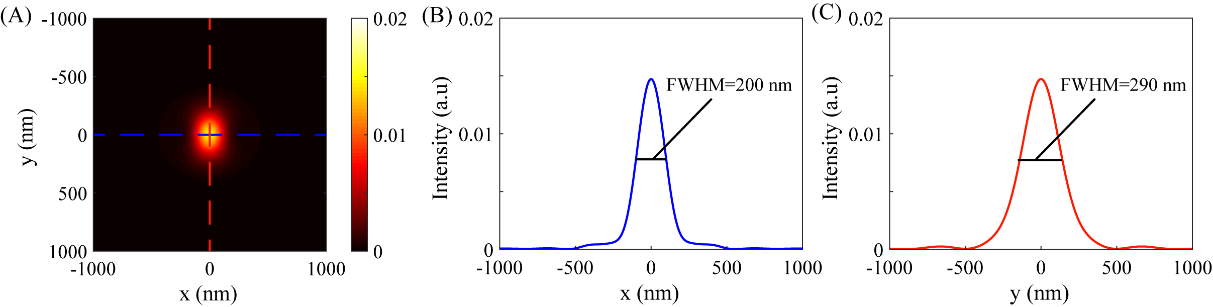


Figure S4. (A) Intensity distributions of the patterns in PR imaged from a circular aperture. (B, C) Corresponding intensity distributions along dashed lines in *x* and *y* directions, respectively.

**S5. The effect of broad spectrum from the mercury lamp used in subwavelength lithography**

The ultraviolet light from the mercury lamp has a broad spectrum with several peaks, and the maximum peak is centered at 365 nm with a FWHM of about 20 nm [7]. Although the optical properties of dielectric, for example, SiO_2_, may not vary significantly across the UV range, the optical properties of Ag and TiO_2_ change evidently. Figure S5 gives the cross-section intensity distributions of the proposed lithography design illuminating with different wavelength lights (360 nm and 370 nm). All the mask patterns are single straight slit with a width of 160 nm, same to the mask used in main text. It is shown that the FWHM of the pattern in PR is 182 nm at the wavelength of 360 nm (Figure S5A, B) and 176 nm at the wavelength of 370 nm (Figure S5C, D). Both larger than 170 nm at the wavelength of 365 nm, which is demonstrated in main text. The realistic dispersive, lossy medium would cause significantly variation in FWHM. Therefore, the FWHM of the pattern is sensitive to the wavelength of lighting source, and the broadband emission from the mercury lamp lead to distinct degraded pattern feature.


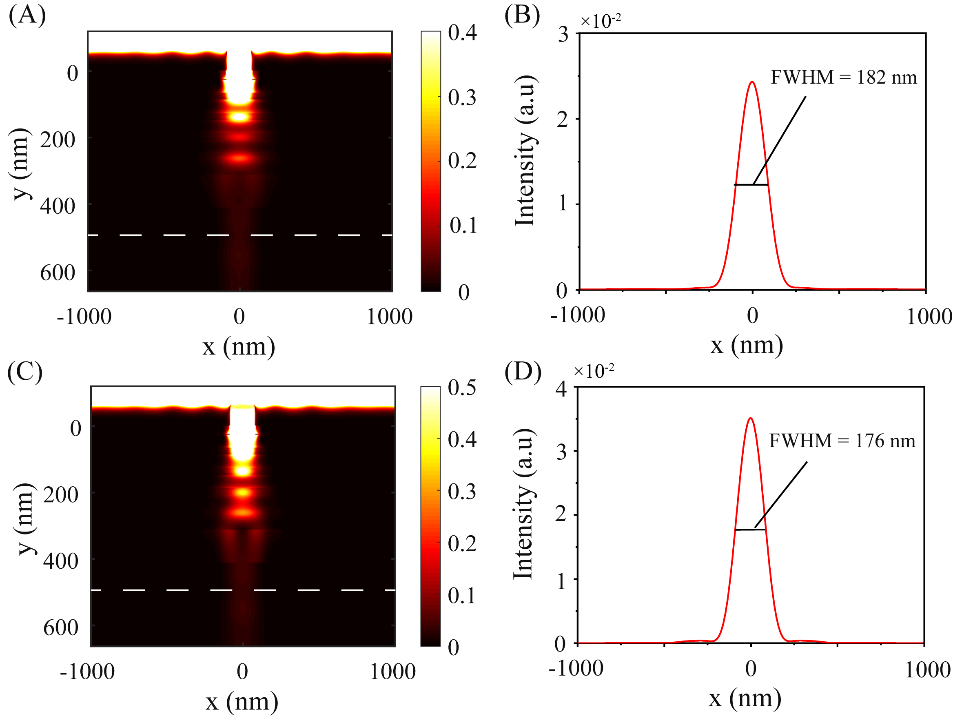


Figure S5. (A) Cross section of normalized intensity distribution at the wavelength of 360 nm. (B) Corresponding intensity distribution along the dashed line in (A). (C) Cross section of normalized intensity distribution at the wavelength of 370 nm. (D) Corresponding intensity distribution along the dashed line in (C).

**S6. Simulations for the control lithography scheme**

In the control group, the NIM multilayer is removed. The light could not be refocused, but diverge gradually as the propagating distance increases, which can be seen in Figure S6A. When the non-polarized light illuminating the straight slit with a width of 160 nm on the mask layer, the transmitted light forms a kind of cylindrical beam. After passing through the 100 nm air WD, the straight line is imaged on the PR surface with an FWHM of ~220 nm, much larger than that in NIM-based condition (~170 nm). Although the pattern could be formed in PR over entire depth (200 nm), the FWHM of the pattern widens as the depth increases, which would be expanded to 350 nm at the PR bottom (Figure S6B, C). This is meaningless for one-to-one photolithography.


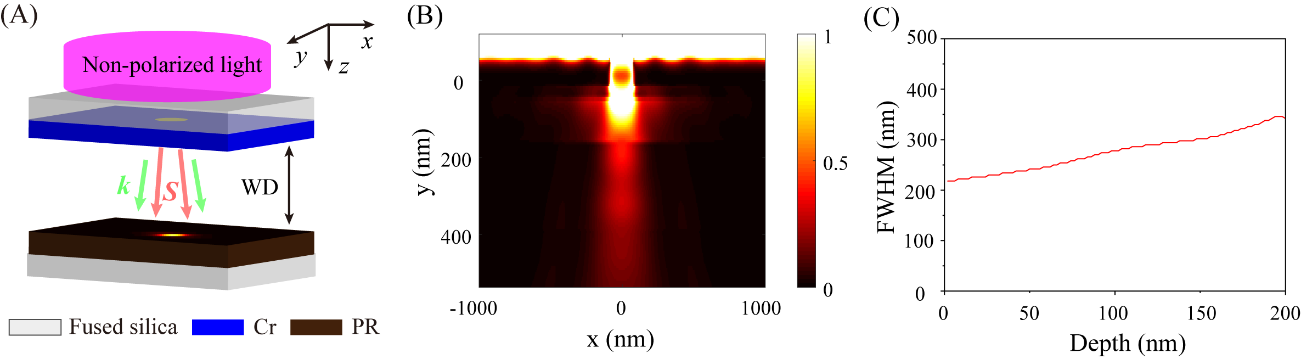


Figure S6. (A) Cross section of normalized intensity distribution of the lithography in control experiment. (B) FWHM variation of the pattern along the transmitted depth in PR.

**S7. Subwavelength rectangles array mask used in NIM-based photolithography**

Figure 6 in main text is primarily to demonstrate the proposed subwavelength lithography system can fabricate typical patterns over large area with a single exposure process. Nonetheless, none of the feature size of the mask patterns are smaller than 365 nm. However, the outstanding performance of the NIM structure is successfully demonstrated by the well-shaped patterns with neat morphologies. If the rectangles array mask is scaled down to subwavelength patterns, the functions of the NIM-based design are remained. Figure S7 is a picture of intensity distribution extracted from the PR layer in lithography system, where the feature size of mask is 300 nm, i.e., 3/4 of that in main text. The surface morphology of the intensity distribution presents clear patterns with different sizes and rotations, and the intensity distribution along the dashed line shows a high contrast, which are in line with the analyses in main text. It proves that producing subwavelength patterns over large area is within the capabilities of the proposed lithography system.


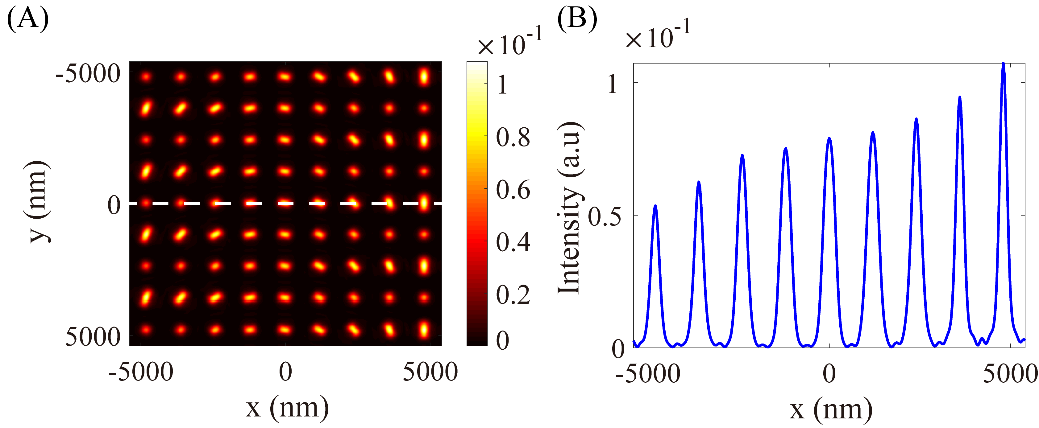


Figure S7. (A) Intensity distributions of the patterns in PR imaged from a subwavelength mask. (B) Corresponding intensity distributions along the dashed lines in (A).

**S8. Annular slits array produced by the NIM-based photolithography**

The NIM-based photolithography system is feasible for producing arbitrary patterns over large area. Figure S8A shows the image of a mask constructed with annular slits array, where the inner diameters of the slits in the same row change from 400 nm to 800 nm with 100 nm increments, but the widths of the slits in the same column increase from 200 nm to 320 nm with 40 nm increments. Figure S8B is the two-dimensional intensity distribution extracted from the middle position of the PR layer. Because the light flux entering the mask grows as the width of the slits increases, the intensity is enhanced accordingly. Thus, there are obvious differences in light intensity among the annular slits array. Usually, sidelobes are intractable problem for this nano-optical devices [8-10]. However, the neat patterns area is obtained here after the lithography process, which indicates the disordered diffraction and intersect interference maybe not happened or affect the imaging process. The experimental results expressed by SEM and AFM pictures clearly present the mask structure is fully imaged in PR with high fidelity (Figure S8C-E). The faulted slits in PR could be ascribed to the defects in mask or NIM multilayer.


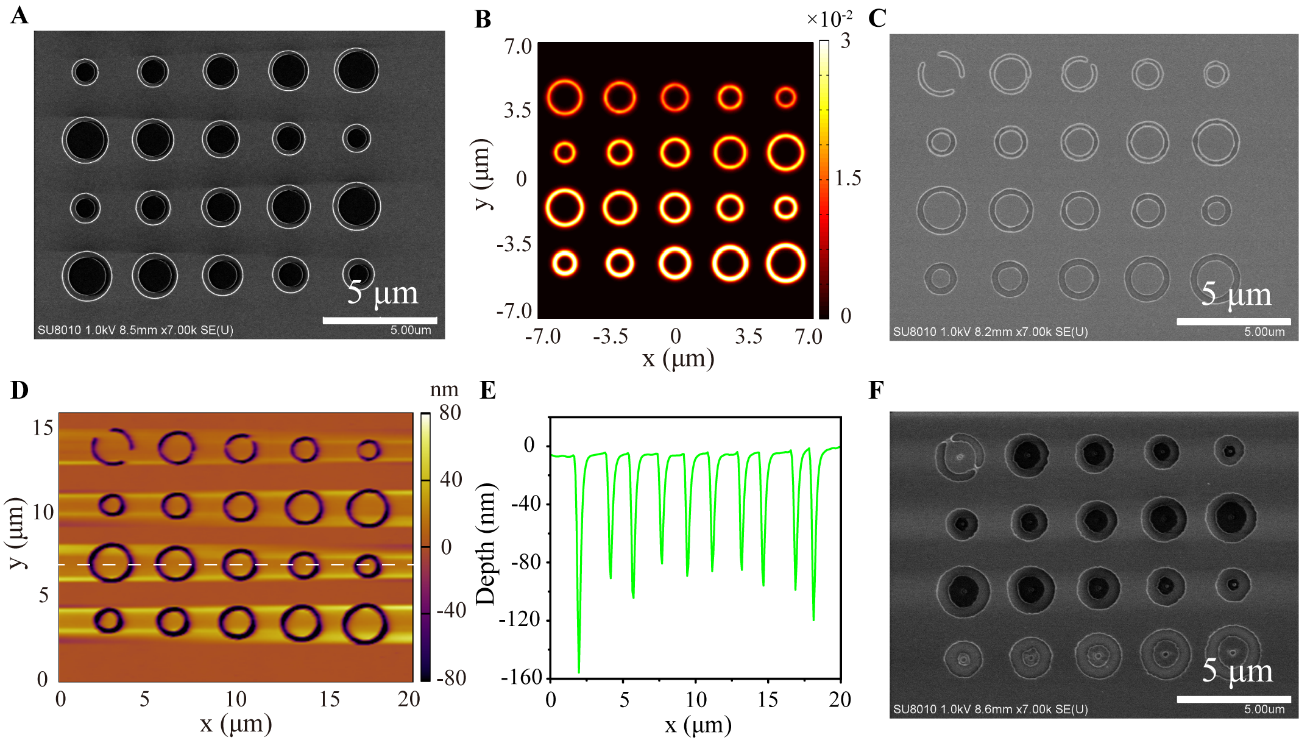


Figure S8. (A) SEM image of a mask constructed with annular slits array. (B) Normalized intensity distributions extracted from the position of 100 nm-depth PR. (C, D) SEM and AFM images of the pattern formed in PR. (E) Corresponding depth profile along the dashed line in (D). (F) SEM image of the PR pattern obtained from control experiment.

In the control experiment, the annular slits are great difference in quality. Even worse, there are tiny rings in the central of the annular slits (Figure S8F). That’s because unwanted interferences are happened for the counterpropagating diffractive waves during the imaging process. Therefore, without the NIM multilayer, the photolithography system with a 100 nm-thick air WD cannot collect the diffracted lights effectively, and the patterns shaped in PR are severely distorted.

**S9. *V*-shaped antennas produced by the NIM-based photolithography**


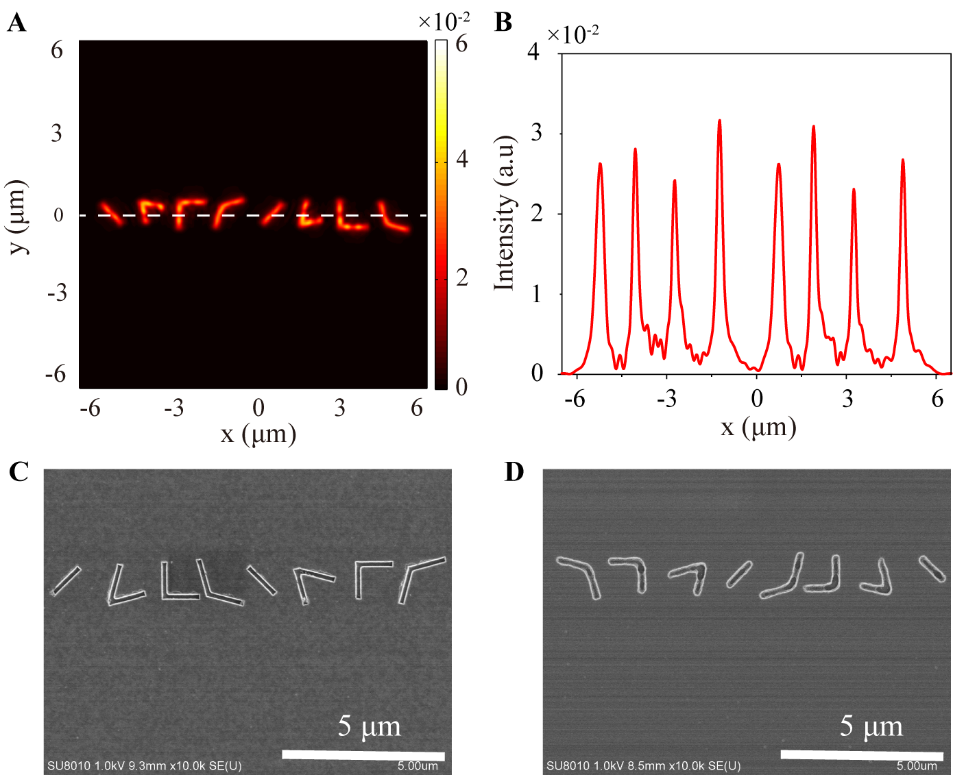


Figure S9. (A) Normalized intensity distributions in *x*-*y* plane extracted from the middle position of the PR. (B) Corresponding intensity distributions along the dashed lines in (A). (C) SEM image of a mask constructed with a group of *V*-shaped antennas. (D) SEM image of the patterns produced by the NIM-based lithography system.

Optical metasurface are attractive research topic in recent years. Typically, *V*-shaped antennas, composed of two rods, are often used to manipulating the wave front effectively [11-13]. The phase could be introduced from 0 to 2π by changing the opening angle of the two rods. However, the fabrication of this subwavelength structure are difficult for traditional lithography technologies. Although electron beam lithography is the mostly method to fabricate this kinds of structures, high-cost and time consuming properties declare it not an optimal way, especially for the nano-patterns over large area. Here, we present a group of *V*-shaped antennas fabricated with the photolithography system proposed in main text. A series of *V*-shaped antennas on the mask is constructed with different opening angle, where the arm length of each antenna is 1.2 μm, the width is 220 nm, and the spacing between the adjacent *V*-shaped antennas is 1.5 μm. Figure S9A is the two-dimensional intensity distribution extracted from the middle position of the PR film. The corresponding field distribution along the white dashed line is shown in Figure S9B. Owing to the negative refraction function of the NIM multilayer, the mask features are successfully reproduced in the PR film with high fidelity (Figure S9C, D). It is proved that the NIM-based lithography system is capable of producing two-dimensional arbitrary patterns, especially for the metasurface-based optical devices.

**References**

[1] T. Xu, A. Agrawal, M. Abashin, K. J. Chau, and H. J. Lezec, “All-angle negative refraction and active flat lensing of ultraviolet light,” *Nature*, vol. 497, pp. 470-474, 2013.

[2] G. Liang, C. Wang, Z. Zhao, et al., “Squeezing bulk plasmon polaritons through hyperbolic metamaterials for large area deep subwavelength interference lithography,” *Adv. Opt. Mater.*, vol. 3, pp. 1248-1256, 2015.

[3] L. Liu, P. Gao, K. Liu, et al., “Nanofocusing of circularly polarized Bessel-type plasmon polaritons with hyperbolic metamaterials,” *Mater. Horiz.,* vol. 4, pp. 290-296, 2017.

[4] E. Verhagen, R. de Waele, L. Kuipers, and A. Polman, “Three-dimensional negative index of refraction at optical frequencies by coupling plasmonic waveguides,” *Phys. Rev. Lett.*, vol. 105, pp. 223901, 2010.

[5] C. Zhang, S. Divitt, Q. Fan, et al., “Low-loss metasurface optics down to the deep ultraviolet region,” *Light: Sci. Appl.*, vol. 9, pp. 1-10, 2020.

[6] T. Siefke, S. Kroker, K. Pfeiffer, et al., “Materials pushing the application limits of wire grid polarizers further into the deep ultraviolet spectral range,” *Adv. Opt. Mater.*, vol. 4, pp.1780-1786, 2016.

[7] D. Shao, and S. Chen, “Surface plasmon assisted contact scheme nanoscale photolithography using an UV lamp,” *J. Vac. Sci. Technol. B*, vol. 26, pp. 227-231, 2008

[8] X. Dai, F. Dong, K. Zhang, et al., “Holographic super-resolution metalens for achromatic sub-wavelength focusing,” *ACS Photonics*, vol. 8, pp. 2294-2303, 2021.

[9] Y. Wang, Q. Fan, and T. Xu, “Design of high efficiency achromatic metalens with large operation bandwidth using bilayer architecture,” *Opto-Electron. Adv*., vol. 4, pp. 20000801-20000815, 2021.

[10] X. Ma, M. Pu, X. Li, Y. Guo, and X. Luo, “All-metallic wide-angle metasurfaces for multifunctional polarization manipulation,” *Opto-Electron. Adv.*, vol. 2, pp. 18002301-18002306, 2019.

[11] R. Blanchard, G. Aoust, P. Genevet, N. Yu, M. A. Kats, Z. Gaburro, and F. Capasso, “Modeling nanoscale V-shaped antennas for the design of optical phased arrays,” *Phys. Rev. B*, vol. 85, p. 155457, 2012

[12] N. Yu, P. Genevet, and M. A. Kats, et al., “Light propagation with phase discontinuities: generalized laws of reflection and refraction,” *Science*, vol. 334, pp. 333-337, 2011.

[13] H. T. Chen, A. J. Taylor, and N. Yu, “A review of metasurfaces: Physics and applications,” *Rep. Prog. Phys.*, vol. 79, p. 076401, 2016.
